# Protective Effects of p38 MAPK Inhibitor SB202190 against Hippocampal Apoptosis and Spatial Learning and Memory Deficits in a Rat Model of Vascular Dementia

**DOI:** 10.1155/2013/215798

**Published:** 2013-12-25

**Authors:** Shen Yang, Guangan Zhou, Hong Liu, Bo Zhang, Juan Li, Ruiting Cui, Yifeng Du

**Affiliations:** ^1^Department of Neurology, Shandong Provincial Hospital, Shandong University, Jinan 250012, China; ^2^Department of Neurology, Taian Central Hospital, Taian 271000, China; ^3^Department of Neurology, Liaocheng Hospital, Liaocheng 252000, China

## Abstract

Vascular dementia (VaD) is a common age-related neurodegenerative disease resulting from chronic hypoxia. In the present study, we examined the protective effects of p38 MAPK inhibitor SB202190 against hippocampal apoptosis and spatial learning and memory deficits in a chronic hypoperfusion rat model of VaD established by permanent bilateral carotid occlusion (2-VO). Sixty rats were randomly divided into sham-operated, VaD model, and VaD plus SB202190 groups (*n* = 20/group). After sham/2-VO surgery, rats were administered 0.1% DMSO (sham-operated and VaD groups) or SB202190 by intracerebroventricular injection. One week after inhibitor/vehicle treatment, hippocampal p38 MAPK phosphorylation was higher in the model group than in the SB202190 group (*P* < 0.01). Compared to the model group, the SB202190 group exhibited significantly shorter escape latencies in the Morris water maze hidden platform trials (*P* < 0.01) and longer times in the original platform quadrant during probe trials (*P* < 0.01). The SB202190 group also showed significantly reduced neuronal apoptosis in the hippocampus compared to VaD model rats (*P* < 0.01) as well as higher (antiapoptotic) Bcl-2 expression and lower (proapoptotic) caspase-3 expression (*P* < 0.01 for both). In conclusion, blockade of the p38 MAPK signaling pathway by SB202190 following permanent 2-OV reduced apoptosis of hippocampal neurons and rescued spatial learning and memory deficits.

## 1. Introduction

Vascular dementia (VaD) is the second most common form of age-related neurological dysfunction in most Western countries after Alzheimer's disease (AD) [1] and may be the most common form in countries with lower rates of AD [2, 3]. The core symptom of VaD is progressive cognitive dysfunction due to cumulative regional brain tissue injury associated with localized cerebrovascular disruptions (microinfarcts or “mini strokes”) [4]. The progressive nature of VaD leads to unremitting and largely irreversible deterioration in quality of life and places a heavy emotional and economic burden on families. In countries with aging populations, prevention and treatment of VaD are a major medical and social priority. Multiple factors increase the risk of CD, including previous stroke, hypertension, and diabetes [5]. Targeting these risk factors can reduce disease incidence or progression but there are no broadly effective treatments that can reverse the deficits of VaD.

The hippocampal formation, including the hippocampus, dentate gyrus, subiculum, and parahippocampus gyrus, is essential for the formation of declarative memories [6]. Specific regions within this mesial temporal lobe structure are highly susceptible to ischemia-reperfusion injury [7]. Activation of the p38 mitogen-activated protein kinase (p38 MAPK) signaling pathway by hypoxia may initiate neuronal apoptosis, leading to the functional deficits of VaD [8]. In the hippocampus, activation of caspase-3 and other markers of mitochondrial apoptosis in response to hyperosmotic stress was blocked by SB202190, a specific inhibitor of p38 MAPK [9]. Conversely, inhibition of p38 MAPK abolished the cytoprotective effects of ischemic preconditioning [10], suggesting that mild p38 MAPK activation may initiate protective responses while overactivation, which may occur during prolonged hypoxia associated with VaD, may activate cell death pathways. Indeed, activation of MAPK is one of the central signal transduction pathways triggering neuronal apoptosis following neural hypoxia and reperfusion [11–13]. Overexpression of Bcl-2 can effectively inhibit caspase-3 activation and apoptosis [14], and p38 MAPK affects apoptosis by regulating Bcl-2 and caspase-3 expression [15]. For example, quercetin, a natural MAPK p38 inhibitor, blocked apoptosis in the hippocampus by preventing Bcl-2 downregulation, Bax upregulation, and caspase-3 activation [16]. Moreover, SB202190 can reduce cerebral ischemia-reperfusion injury [17].

No previous study has examined the effect of SB202190 for sustaining hippocampus-dependent spatial memory or the effects of this inhibitor on the expression of apoptotic regulators under chronic ischemia. To these ends, we examined the effects of SB202190 on hippocampal neuron apoptosis, Bcl-2 and caspase-3 expression, and p38 MAPK phosphoactivation in a rat model of VaD and tested whether p38 MAPK inhibition can rescue deficits in the hippocampus-dependent Morris water maze test of spatial learning and memory. Our results indicate that blockade of the p38 MAPK signaling pathway can indeed protect hippocampal neurons against chronic ischemic injury and rescue spatial learning and memory deficits, at least in part, by suppressing caspase-3-dependent apoptosis.

## 2. Materials and Methods

### 2.1. Reagents

Rabbit anti-rat Bcl-2, caspase-3 monoclonal antibodies, SB202190, FITC-conjugated goat anti-rabbit secondary antibody, p-p38 MAPK rabbit anti-rat polyclonal antibody, horseradish peroxidase- (HRP-) conjugated goat anti-rabbit IgG, enhanced chemiluminescence (ECL), bicinchoninic acid (BCA) protein assay, and lysis buffer were purchased from Santa Cruz (CA, USA). *β*-Actin was purchased from Yanjing Biological Research Technology Co. (Shanghai, China), an *in situ* apoptosis detection kit from Roche (USA), and an SABC-FITC immunofluorescence assay kit from Boster (Wuhan, China).

### 2.2. Experimental Devices

A Morris water maze (DMS-2 type) was obtained from the Chinese Academy of Medical Sciences, a rat stereotaxic apparatus (Jiangwan type I-C) from Huaibei Zhenghua Biologic Apparatus Facilities (Anhui, China), a Radiance 2100 type laser scanning confocal microscope and Gel Doc gel imaging analysis system from Bio-Rad (USA), and a freezing microtome from Leica (CM1900 type, Leica, Germany).

### 2.3. Animals

Specific pathogen-free (SPF) male Wistar rats (*n* = 60, three month old, 250 ± 10 g) were purchased from the Experimental Animal Center of Shandong University School of Medicine (China) (Certificate no. SCXK Lu 20090001) and maintained at the Animal House of Taishan Medical University (Shandong, China). The rats were housed at 20–25°C and 50% ± 5% humidity with ad libitum access to food and water under a 12 : 12 h light/dark cycle for two weeks before experiments. All procedures and animal experiments were approved by the Animal Ethical Committee of Taishan Medical University and conducted in accordance with all state regulations.

### 2.4. Animal Grouping

The 60 Wistar rats were randomly assigned to the sham-operated group, the VaD model group, and the SB202190 group (20 animals each) using a random number table. The VaD rat model (*n* = 40) was established by separating and ligating the bilateral carotid artery via two-vessel occlusion (2-VO). For animals of the sham-operated group (*n* = 20), the bilateral carotid artery was separated using the same methods but without ligation. After recovery, animals of the SB202190 group received intracerebroventricular (ICV) injection of SB202190 (dissolved in 100% DMSO and then diluted in normal saline (NS) for a final concentration of DMSO of 0.1%) and both the VaD model and sham-operated groups received ICV injection of equal volume 0.1% DMSO. In each group, eight rats were examined in the Morris water maze to assess spatial learning and memory, six rats were sacrificed and brain sections were prepared for TUNEL staining and Bcl-2/caspase-3 immunohistochemistry, and six rats were sacrificed and tissue homogenates were prepared for Western blot assay of phospho-p38 MAPK expression.

#### 2.4.1. Permanent Bilateral Carotid Occlusion (2-VO)

Following 12 h of fasting and 4 h without water, animals were anesthetized by intraperitoneal (IP) injection of a 10% chloral hydrate solution at 0.35 mL/100 g body weight. The injected dose of chloral hydrate was increased when necessary. After loss of the righting reflex, animals were fixed in the supine position on a rat board. An incision was made along the midline of the neck, followed by careful blunt dissection of muscle and connective tissues. The bilateral common carotid artery was isolated and ligated with silk sutures [13]. If normal respiration and heartbeat were maintained, the skin and muscle layers were sutured. Gentamicin (80,000 units) was applied to the surgical wound by topical spray and intramuscular injection for three consecutive days. During recovery, the animals were housed and fed in single cages to prevent infection.

#### 2.4.2. Intracerebroventricular Injection

All animals were administered 0.1% DMSO or SB202190 immediately after sham or 2-VO surgery by ICV injection. Briefly, after anesthesia by IP injection of a 10% chloral hydrate (0.35 mL/100 g body weight), the animal was secured within a Jiangwan I-C rat stereotaxic frame. The site for ICV injection was 0.8 mm caudal to bregma and 1.5 mm to the right of the midline. A hole was drilled horizontally with an electric drill and 5 *μ*L of 0.1% DMSO or 10 *μ*mol/L SB202190 solution was injected using a microinjector. The injection was completed in 4 min and the needle kept in position for an additional 2 min. One week after ICV injection, animals were examined in the Morris water maze or sacrificed and brain tissues were prepared for TUNEL staining, immunofluorescence, and Western blot.

#### 2.4.3. Morris Water Maze Test

The Morris water maze setup consisted of a circular pool (diameter: 100 cm, height: 50 cm; wall: blank), a hidden platform, and an overhead video recording system. The pool was arbitrarily divided into northeast, southeast, southwest, and northwest quadrants. Each day, water was poured into the pool to submerge a cylindrical platform at the center of a chosen quadrant 2 cm below the surface. The water was made cloudy using black polyethylene particles and maintained at 23°C ± 2°C. The pool was surrounded by black curtains and maintained in the same position, allowing the rats to locate the platform. A digital camera was placed approximately 1 m above the center of the pool to record swim paths. The video signals were exported to a computer and analyzed using an automatic image acquisition and analysis system (Drug Research Institute of Chinese Academy of Medical Sciences). Hidden platform trials were conducted for three consecutive days and escape latencies recorded as an index of spatial learning. Memory for platform location was tested on the fourth day by a probe test in which the platform was removed and time spent in the original platform quadrant was recorded [18].

#### 2.4.4. TUNEL Staining

In each group, six rats were decapitated and hippocampal tissue was isolated and immediately frozen in liquid nitrogen. Frozen sections were prepared at approximately 10 *μ*m and fixed in freshly prepared 4% paraformaldehyde at room temperature for 30 min. After washing with PBS, a blocking agent (0.3% H_2_O_2_ methanol solution) was added dropwise and slices were incubated at room temperature for 30 min. Slices were then washed in PBS again and permeabilized by drop-wise addition of ice-cold 0.1% Triton X-100 in 0.1% citric acid solution for 2 min. Slices were washed with PBS twice, excess liquid was removed using tissue paper, and 50 *μ*L of TUNEL reaction mixture was added drop-wise, followed by incubation in a wet box at 37°C for 60 min. Finally, the slices were washed three times with PBS, mounted with a 1 : 1 glycerol PBS solution, and examined under a confocal laser scanning microscope. Images were taken using the Lasersharp 2000 software package. For each animal, slices were randomly examined and the average number of TUNEL-positive cells was determined from 30 random fields of view [19].

#### 2.4.5. Immunofluorescence

Hippocampal tissue samples were isolated and immediately frozen in liquid nitrogen. Frozen sections were prepared at approximately 8 *μ*m on a cryostat microtome and then fixed in freshly prepared 4% paraformaldehyde at room temperature for 30 min. After antigen retrieval, 0.4% Triton X-100 and 1% bovine serum albumin were added drop-wise, followed by incubation at room temperature for 1 h. Goat serum blocking solution was added drop-wise and slices were incubated for 5 h. After removing excess blocking solution, a solution of 1 : 200 rabbit anti-rat Bcl-2 or caspase-3 monoclonal antibody in 0.01 mol/L PBS was added drop-wise and slices were incubated at 4°C overnight. Thereafter, the primary antibody was washed off with PBS (three times), and the FITC-conjugated secondary antibody was added. The immunolabeled slices were mounted with 1 : 1 diluted glycerol in PBS and FITC-positive cells were detected by confocal laser scanning microscopy. Both fluorescence intensity and the number of FITC-labeled cells were determined in 30 random fields of view [20].

#### 2.4.6. Western Blotting

Hippocampal tissue was isolated and homogenized in lysis buffer (in mM: 50 Tris-Cl pH 7.5, 150 NaCl, 40 KF, 5 EDTA, 5 EGTA, 1 orthovanadate, and 1 PMSF with 0.1% SDS, 0.1% aprotinin, 0.1% sodium deoxycholate, and 0.5% NP-40) on ice. The homogenate was centrifuged (13,000 ×g, 15 min, 4°C) and the total supernatant protein was measured by BCA assay. Then, 50 *μ*g of protein per gel lane was separated by 10% SDS-PAGE and proteins were transferred to nitrocellulose membranes (Santa Cruz). Membranes were blocked by a one-hour incubation in TBS containing 10% skim milk powder and 0.05% Tween 20 (TBST). The blocked membranes were incubated in primary antibody (1 : 1000 p-p38 MAPK rabbit anti-rat polyclonal antibody or 1 : 2000 *β*-actin as an internal reference) at 4°C for 2 h and then with the secondary antibody (HRP-conjugated goat anti-rabbit IgG) at room temperature for 1 h. Immunolabeling was then visualized by ECL. The relative intensity of each protein band was measured by the Gel Doc gel imaging analysis system. Results are presented as the optical density ratio of p-p38 MAPK and *β*-actin.

#### 2.4.7. Statistical Analyses

All statistical tests were conducted using SPSS15.0 software (SPSS Inc., Chicago, IL, USA). Data are expressed as means ± standard deviation (SD). Statistical significance was evaluated by one-way analysis of variance (ANOVA) with Tukey's test for post hoc analysis. A *P* < 0.05 was considered statistically significant.

## 3. Results

### 3.1. Effect of Chronic Ischemia on p38 MAPK Phosphoactivation in the Hippocampus of VaD Rat Model

**Figure 1:**
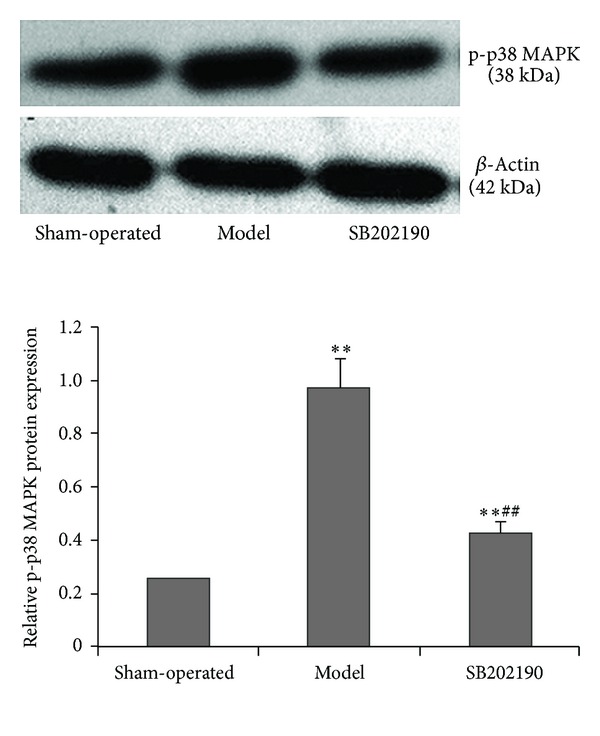
Effect of the p38 MAPK inhibitor SB202190 on expression of p-p38 MAPK in the hippocampus of a vascular dementia (VaD) rat model. Expression of p-p38 MAPK was determined by Western blot and protein expression normalized to *β*-actin. The data are expressed as means ± standard deviation (SD) (*n* = 6); **P < 0.01 versus sham-operated group, ^##^ *P* < 0.01 SB202190 group versus model group.

Rats were subjected to either permanent bilateral carotid artery occlusion (VaD model group, *n* = 20; SB202190 p38 MAPK inhibitor group, *n* = 20) or sham operation (*n* = 20) and then immediately administered 0.1% DMSO (sham-operated and VaD model groups) or SB202190 by ICV injection. One week after ICV injection, we observed significant differences in hippocampal p-p38 MAPK expression levels among the treatment groups (*P* < 0.01), with significantly higher expression in the VaD model group compared to the sham-operated group (0.974 ± 0.110 versus 0.254 ± 0.021; *P* < 0.01). Compared to the model group, p-p38 MAPK expression was significantly reduced by SB202190 (0.432 ± 0.039) (*P* < 0.01) (Figure 1).

### 3.2. Protective Effects of SB202190 against Spatial Learning and Memory Deficits in a Rat Model of VaD

One week after ICV injection, hippocampus-dependent spatial learning was examined by the MWM hidden platform test. On each of the three testing days, escape latency was significantly longer in the model group than that in the sham-operated group (all *P* < 0.01), indicative of hippocampus-dependent spatial learning deficits. Latencies were significantly shorter in rats injected with SB202190 compared to the VaD model group on each day of testing (all *P* < 0.01). Over the 3 days of training, mean escape latency in the sham-operated rats decreased from 24.17 ± 4.12 s to 16.83 ± 3.77 s (*P* < 0.01), indicating spatial learning, whilst latency in the VaD model group was not significantly reduced by training (from 82.71 ± 8.27 s to 77.74 ± 6.33 s, *P* > 0.05). Escape latencies in the SB202190 group decreased from 48.72 ± 7.01 s to 40.34 ± 2.46 s (*P* < 0.05), indicating spatial learning improvement (Table 1).

On day 4, the hidden platform was removed from the MWM in order to conduct a probe test of spatial memory. The time spent in the original platform quadrant was significantly longer in the VaD model group than in the sham-operated group (*P* < 0.01), while target quadrant time was lower in the SB202190 group than in the VaD model group (*P* < 0.01), indicating partial rescue to spatial memory (Table 2). In contrast, there was no difference in swim speed among groups, indicating that motor impairments did not account for these differences in training or probe test performance. Thus, prolonged latencies in the training trial are indicative of disrupted spatial learning in VaD model rats, while reduced time in the target quadrant is indicative of impaired hippocampus-dependent spatial memory. Moreover, reduced escape latency and longer target quadrant time in the SB202190 group compared to the model group indicate that blockade of p38 MAPK signaling partially preserves spatial learning and memory during chronic ischemia.

### 3.3. SB202190 Reduced Apoptosis of Hippocampal Neurons in a Rat Model of VaD

**Figure 2:**
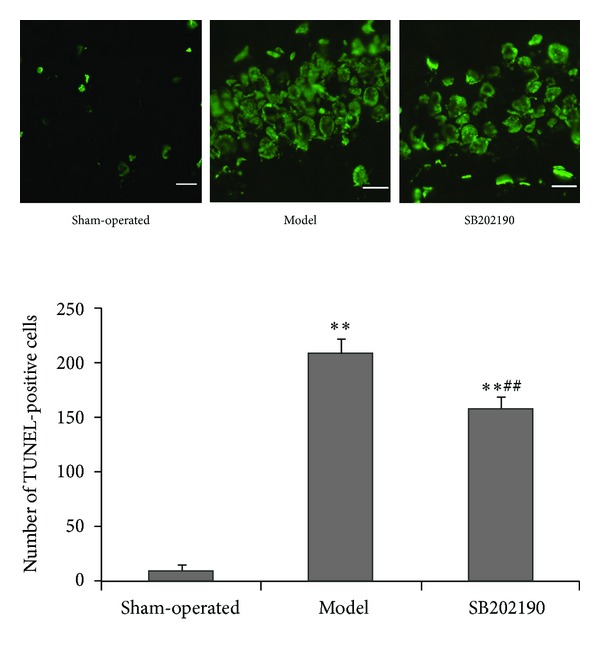
Effect of SB202190 on neural apoptosis in the hippocampus of the VaD rat model. Neural apoptosis was assessed by TUNEL (magnification ×40). Data are expressed as means ± SD (*n* = 6); **P < 0.01 versus sham-operated group, ^##^ *P* < 0.01 SB202190 group versus model group.

One week after ICV injection, TUNEL-positive (green fluorescent) cell numbers were significantly increased in model and SB202190 groups compared to the sham-operated group (both *P* < 0.01), but the number of TUNEL-positive cells was significantly lower in the SB202190 group (*P* < 0.01) (Figure 2). Thus, inhibition of p38 MAPK protected hippocampal neurons against chronic ischemia-induced apoptosis.

### 3.4. Effects of SB202190 on Bcl-2 and Caspase-3 Expressions in the Hippocampus of VaD Rat Model

**Figure 3:**
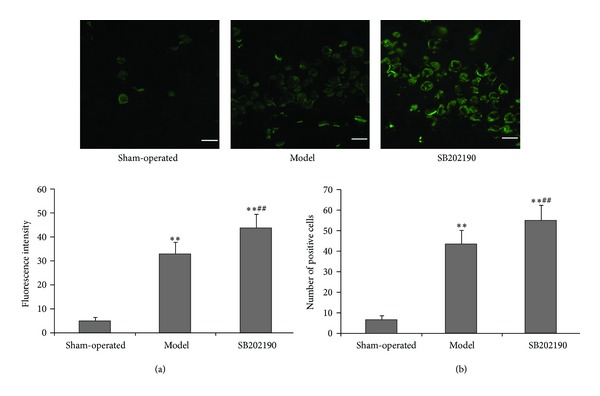
Effect of SB202190 on expression of Bcl-2 in the hippocampus of the VaD rat model. Expression of Bcl-2 was determined by immunofluorescence staining (mMagnification ×40). Green: FITC. (a) Fluorescence intensity; (b) number of positive cells. The data are expressed as means ± SD (*n* = 6); **P < 0.01 versus sham-operated group, ^##^ *P* < 0.01 SB202190 group versus model group.

One week after ICV injection, only faint Bcl-2 immunofluorescence was observed in hippocampal slices prepared from sham-operated rats; by comparison, the Bcl-2 immunofluorescence intensity was significantly higher in slices from VaD model and SB202190 groups but was significantly higher in the SB202190 group (all *P* < 0.01) (Figure 3(a)). These differences in immunofluorescence intensity were paralleled by changes in Bcl-2-positive cell number, which was significantly higher in hippocampal slices from SB202190 group rats from than VaD model group rats (*P* < 0.01) (Figure 3(b)), suggesting that inhibition of p38 MAPK may reduce apoptosis by enhancing expression of this antiapoptotic protein.

**Figure 4:**
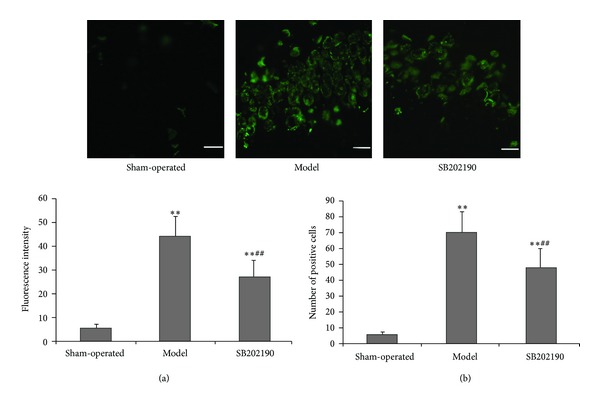
Effect of SB202190 on expression of caspase-3 in the hippocampus of the VaD rat model. Expression of caspase-3 was determined by immunofluorescence staining. (magnification ×40). Green: FITC. (a) Fluorescence intensity; (b) number of positive cells. Data are expressed as means ± SD (*n* = 6); **P < 0.01 versus sham-operated group, ^##^ *P* < 0.01 SB202190 group versus model group.

One week after ICV injection, only faint caspase-3 immunofluorescence was detected in hippocampal slices from sham-operated rats compared to VaD model group rats, indicating that chronic ischemia may activate the mitochondria-dependent apoptosis pathway. However, caspase-3 immunofluorescence was significantly lower in slices from the SB202190 group compared to the model group (*P* < 0.01) (Figure 4(a)), and again this increase in immunofluorescence was paralleled by an increase in caspase-3-positive cell number (*P* < 0.01) (Figure 4(b)).

## 4. Discussion

Vascular dementia is a major cause of senile dementia, accounting for 30%−50% of cases by region [3]. Moreover, VaD is often comorbid with AD, and these two forms of dementia may act synergistically to damage neural tissue and impair cognition [1]. The hippocampus is highly sensitive to ischemia and hypoxia; indeed, it usually sustains the greatest injury as evidenced by infarct volume during chronic cerebral hypoperfusion [21]. Chronic cerebral hypoperfusion is the primary cause of VaD. The present study established a chronic cerebral hypoperfusion model by bilateral carotid artery ligation and demonstrated severe disruption of hippocampus-dependent spatial learning and memory, strong phosphoactivation of p38 MAPK, and enhanced activation of caspase-3-dependent apoptosis. Most importantly, these neurocellular and cognitive defects were partially reversed by blockade of p38 MAPK. Thus, activation of p38 MAPK by chronic ischemia activates apoptosis in the hippocampus, which ultimately disrupts spatial learning. Duarte et al. [22] also found p38 MAPK activation during ischemia-reperfusion, suggesting that p38 MAPK is also a potential therapeutic target for stroke. Thus, neuroprotection by p38 MAPK inhibition is a possible therapeutic strategy for preserving neuronal viability and cognition in patients with cerebrovascular disorders.

A myriad of animal models of neurovascular disease and acute stroke have been established to elucidate pathogenic mechanisms and identify possible neuroprotective strategies. Such models have helped reveal glutamate-mediated excitotoxicity, calcium dysregulation, oxidative stress, acidosis, edema, mitochondria-dependent apoptosis, proteolysis, and inflammation as major mutually reenforcing pathogenic processes leading to neural damage during ischemia-reperfusion [23]. Conversely, few compounds identified as neuroprotectants in animals have proven effective in clinical practice [24], so these models either do not replicate human stroke pathogenesis, are more resistant to ischemia, or both. The first chronic cerebral hypoperfusion model used stents of different sizes to partially occlude cerebral blood flow in mice [25] and resulted in delayed infarct development in both white and gray matter. In the current study, we used total occlusion as the rat brain is generally less sensitive to ischemia compared to humans, at least in most experimental conditions [23, 26, 27]. First, rats used in stroke modeling are almost always young, healthy, genetically homogenous SPF males, while human stroke patients are usually elderly and have multiple comorbidities and risk factors [27]. Second, brain size, length and structure of perforating arteries, and gray to white matter ratio (lower in humans) influence both stroke resistance and the penetration of potentially neuroprotective drugs [26]. Third, the temporal window for reperfusion or neuroprotectant treatment may not be achieved in humans [23], while administration of possible neuroprotectants in studies such as ours is predetermined. Other possible reasons for differences in human ischemic tolerance included difference in astroglia [28], which can be either neuroprotective or facilitators of neuronal death and impeders of recovery under specific conditions [29]. Finally, hypoperfusion results in mild hypothermia, which is also broadly neuroprotective [23]. Considering that even strains of rats show marked differences in ischemic vulnerability [30], there are obviously a multitude of additional factors contributing to this differential stroke sensitivity between rats and humans. In light of these considerations, potential therapies should ultimately be tested in both young and older rats as well as in rats genetically prone to spontaneous stroke. Furthermore, multiple stroke induction mechanisms should be tested. Now that we and others [31] have identified p38 MAPK inhibitors as potential therapeutics, such extensive studies may be warranted.

Much is known of the mechanism leading from p38 MAPK activation to neurological disease. p38 MAPK is a member of the larger MAPK family involved in pathophysiological processes like inflammation, stress responses, and hyperproliferation [32, 33]. Once activated by upstream phosphorylation, p38 MAPK further activates downstream transcription factors, MAPK-activated protein kinases, and caspases. SB202190 is a pyridinyl imidazole derivative that can effectively and selectively inhibit p38 MAPK activation by preventing its binding to ATP. Fernandez et al. [34] demonstrated that SB202190 can alleviate learning and memory impairment in rats with chronic cerebral hypoperfusion-ischemia, a state resembling VaD. The present study was designed to confirm these results and elucidate the underlying molecular mechanisms for this effect.

The escape latency to the hidden platform in the MWM was significantly shorter and the time spent in the platform (target) quadrant is longer in the SB202190-pretreated VaD group compared to the vehicle-treated VaD group. Zhao et al. [35] reported that p38 MAPK inhibition improved learning and memory in rats subjected to ischemia-reperfusion by regulating the expression of pro- and antiapoptotic factors. Similarly, expression of antiapoptotic Bcl-2 was enhanced in SB202190-treated VaD rats while caspase-3 expression was reduced compared to vehicle-treated VaD rats. Apoptosis is strictly controlled by multiple gene families, including the Bcl-2 and caspase families, as well as by c-myc and p53. The reciprocal regulation of apoptosis by Bcl-2 and caspase-3 is well described [36]. Increasing Bcl-2 expression in brain can reduce cerebral infarct volume by protecting neurons surrounding the ischemic focus (penumbra) [37]. Yang et al. found that ICV injection of a caspase-3 inhibitor reduced infarct area and neural apoptosis [38]. Bcl-2 is an upstream inhibitor of capsase-3, the primary effector of apoptosis. After stress-induced phosphoactivation, p38 MAPK activates gene expression patterns that can alternately induce proliferation, differentiation, cytokine synthesis, or caspase-3-dependent apoptosis. Chen et al. [17], and Liu et al. [39] showed that the learning and memory capacities of VaD rats were closely related to p38 MAPK expression. Results from the present study confirmed these findings and further demonstrate that the p38 MAPK pathway regulates expression of Bcl-2 and caspase-3. SB202190 inhibits neuronal apoptosis at least in part by sustaining overexpression of Bcl-2, thereby inhibiting pathological processes leading to caspase-3 activation, such as activation of the mitochondrial permeability transition pore (mPTP), loss of inner membrane potential, and cytochrome c release.

In VaD, the protective effects of SB202190 against hippocampal neuronal apoptosis and spatial learning and memory deficits were likely due in part to suppression of the caspase-3 apoptotic pathway by Bcl-2 upregulation. While many of these protective effects were moderate, it is important to reemphasize that multiple interacting pathogenic processes contribute to stroke-induced neurodegeneration. Thus, inhibition of p38 MAKP may benefit stroke only when used in combination with other agents, such as thrombolytics, antioxidants, calcium chelators, and anti-inflammatories [24]. It is also clear that the p38 MAPK signaling pathway has both beneficial and deleterious effects on the nervous system during stress. Identifying those conditions in which p38 MAPK contributes to neuronal damage may allow for the judicious use of nontoxic inhibitors for treatment of neurological diseases, such as VaD and acute stroke.

## Figures and Tables

**Table 1:** Comparison of escape latencies in Morris water maze learning trials among treatment groups.

Groups	Day 1	Day 2	Day 3
Sham-operated	24.17 ± 4.12	21.59 ± 5.13	16.83 ± 3.77^ΔΔ^
Model	82.71 ± 8.27**	80.36 ± 9.65**	77.74 ± 6.33**
SB202190	48.72 ± 7.01^∗∗##^	42.41 ± 4.06^∗∗##^	40.34 ± 2.46^∗∗##Δ^

*Note*. Data are expressed as means ± standard deviation (SD) (*n* = 8); **P < 0.01 versus sham-operated group, ^##^ *P* < 0.01 SB202190 group versus model group, ^Δ^ *P* < 0.05, ^ΔΔ^ *P* < 0.01 versus Day 1.

**Table 2:** Comparison of target quadrant time and swim speed during memory probe trials among treatment groups.

Groups	Time spent in the original platform quadrant (s)	Speed (cm/s)
Sham-operated	35.21 ± 3.78	13.46
Model	18.67 ± 5.39**	12.19
SB202190	26.45 ± 4.66^∗∗##^	12.28

*Note*. Data expressed as means ± SD (*n* = 8); **P < 0.01 versus sham-operated group, ^##^ *P* < 0.01 SB202190 group versus model group.
